# Serum triglyceride levels and related factors as prognostic indicators in COVID‐19 patients: A retrospective study

**DOI:** 10.1002/iid3.469

**Published:** 2021-07-08

**Authors:** Peng Zhong, Zhenzhou Wang, Zhe Du

**Affiliations:** ^1^ Department of Ultrasound, Beijing Friendship Hospital Capital Medical University Beijing China; ^2^ Trauma Center, National Center for Trauma Medicine, Key Laboratory of Trauma and Neural Regeneration Peking University People's Hospital Beijing China

**Keywords:** COVID‐19, IL‐10, inflammation, macrophage, prognosis, serum ferritin, triglycerides

## Abstract

The role of triglycerides (TG) in coronavirus disease (COVID‐19) is controversial. The objective of this study was to explore the relationship between TG levels and prognosis in COVID‐19 patients and investigate the factors that affect TG. COVID‐19 patients were divided into normal or high TG level groups. Their demographic data, medical history, signs and symptoms, laboratory results, and final clinical results were analyzed retrospectively. A total of 174 patients were included. TG level was 1.6 (interquartile range [IQR]: 1.1‒2.1) mmol/L for all patients; 2.2 (IQR: 1.8‒2.7) mmol/L and 1.1 (IQR: 1.0–1.3) mmol/L in the high TG and control groups, respectively. Overall, 29 patients (16.7%) died during hospitalization, including 19 (23.1%) in the high TG group and 10 (11.5%) in the control group (absolute survival difference, 2.5% (95% confidence interval [CI], 1.2%‐5.1%), log‐rank *χ* ^2^ = 5.7, and *p* = .017). Serum ferritin, C‐reactive protein (CRP), lactate dehydrogenase (LDH), and interleukin‐10 (IL‐10) levels were significantly higher in the high TG group compared to the control group. Pearson correlation analysis showed that TG was positively correlated with fasting blood glucose, leukocyte, serum ferritin, LDH, CRP, and IL‐10 levels. Multiple regression showed that serum ferritin and IL‐10 levels affected the TG level (*R* ^2^ = .095). The TG level in COVID‐19 patients is correlated to serum ferritin and IL‐10 levels, which reflects the activation of macrophages. It is suggested that COVID‐19 patients be monitored for elevated TG as both a prognostic indicator and potential therapeutic target for COVID‐19.

## Graphical abstract

The triglycerides (TG) level in coronavirus disease (COVID‐19) patients is correlated to serum ferritin and IL‐10 levels, which reflects the activation of macrophages. It is suggested that COVID‐19 patients be monitored for elevated TG as both a prognostic indicator and potential therapeutic target for COVID‐19.

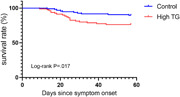

## 1 BACKGROUND

Severe acute respiratory syndrome coronavirus 2 (SARS‐CoV‐2), the causative agent of coronavirus disease (COVID‐19), is highly contagious and has resulted in a global pandemic that is causing significant damage to public health. The elderly and people with diabetes, cardiovascular disease, and low immune function are more likely to have severe COVID‐19.^1^

Some COVID‐19 patients have high triglyceride (TG) levels.^2^, ^3^ A comprehensive untargeted metabolomic and lipidomic approach has shown that TG levels are well‐correlated to the severity of the disease.^4^ Research by Ehrlich et al. showed that elevated lipid metabolism may underline aspects of COVID‐19 pathogenesis, as fenofibrate reverses the metabolic changes induced by SARS‐CoV‐2, blocking viral replication.^5^ On the contrary, research by Wang et al. showed a negative correlation between serum TG levels and the time from disease onset to positive‐to‐negative transmission (PTNT) in nucleic acid tests. This prompted the authors to suggest that patients with COVID‐19 receive high‐fat diets to increase serum TG levels and decrease the PTNT.^6^

However, the role of TG in COVID‐19 is still unclear. Therefore, we retrospectively studied the characteristics of patients with different TG levels and analyzed the factors that affect TG levels, to preliminarily understand the pathophysiological mechanism of TG in COVID‐19.

## 2 MATERIALS AND METHODS

### 2.1 Patients

Overall, 186 patients were diagnosed with COVID‐19 from February 9, 2020 to February 29, 2020 in three wards of Wuhan Tongji Hospital, China. All patients were confirmed positive for SARS‐CoV‐2 nucleic acid by quantitative reverse‐transcription polymerase chain reaction. To avoid the effect of drugs on TG levels, 12 patients were excluded (one case was treated with hydroxychloroquine for chronic systemic lupus erythematosus, six cases were treated with statins, and six cases were treated with lopinavir and ritonavir tablets). Thus, 174 patients were included in the study. According to “The Third Report of the National Cholesterol Education Program Expert Panel on Detection, Evaluation, and Treatment of High Blood Cholesterol in Adults (Adult Treatment Panel III) final report,”^7^ patients with TG ≥ 1.7 mmol/L were included in the study and divided into two groups, high TG group (*n* = 78) and control group (*n* = 96). Information for all patients was retrospectively collected through the electronic medical record system, and included demographic characteristics, medical history, symptoms and signs, laboratory test results, SaO_2_ at the time of admission without oxygen, and final clinical outcomes. Laboratory examination during the course of the disease showed leukocyte and lymphocyte levels were at their minimum values and TG, serum ferritin, C‐reactive protein (CRP), interleukin (IL), tumor necrosis factor (TNF)‐α, lactate dehydrogenase (LDH), erythrocyte sedimentation rate, creatine kinase, and fasting blood glucose levels were at their maximum values. Laboratory tests were carried out early morning in a fasted state, while the examination frequency was subject to disease changes throughout hospitalization. A total of 132 patients were tested for HbA1c, and no glucocorticoid drugs were used before the test. The diagnosis and treatment of COVID‐19 were based on the “New Coronavirus Pneumonia Diagnosis and Treatment Program (Trial Seventh Edition)”^8^ by the National Health Commission of the People's Republic of China. Comorbidities were treated according to the corresponding guidelines. Nonsurvivors and a small number of survivors used antibacterial drugs and glucocorticoids. All patients had good compliance with the treatment. All patients were followed up until April 6, 2020. This study was approved by the Ethics Committee of Peking University People's Hospital (No. 2020PHB110‐01), China.

### 2.2 Statistical analyses

Categorical variables are presented as frequencies and percentages, and continuous variables are presented as median and interquartile range (IQR). One‐way analysis of variance (ANOVA) was used to calculate the difference between groups for continuous variables that fit normal distribution, and non‐parametric tests were used for non‐normally distributed continuous variables. The *χ* ^2^ test was applied to categorical variables. The log rank test was used for survival analysis to test the null hypothesis of no difference between the two groups. Simple regression and multiple linear regression were used to analyze the factors that affect TG. A value of *p* < .05 was considered statistically significant. All statistical analyses were performed using SPSS 24.0 (IBM).

## 3 RESULTS

The demographic baseline characteristics of the patients are shown in Table 1 and laboratory test data are shown in Table 2. All 174 patients enrolled in the study tested positive for SARS‐CoV‐2 RNA. The patients had a median age of 66 (IQR: 54.7–72.0, range: 24–95) years, and a male to female ratio of 88/86. A total of 117 patients (67.2%) had comorbidities, among which hypertension (46.0%) was the most common, followed by diabetes (29.3%), and cardio‐cerebrovascular disease (17.2%). Eleven (6.3%) patients who received chronic hemodialysis for Stage 5 chronic kidney disease were infected with SARS‐CoV‐2 and received intermittent renal replacement therapy at the bedside (Table 1).

**Table 1:** Demographics and baseline characteristics of COVID‐19 patients

	Number (%)			
Variables	Total (*n* = 174)	High TG (*n* = 78)	Control (*n* = 96)	*p*‐value
Age in years, median (IQR)	66.0 (54.7–72.0)	63.0 (50.0–69.0)	67.0 (57.0–73.0)	.151
Sex				
Male	88 (50.6)	35 (44.9)	53 (55.2)	.223
Female	86 (49.4)	43 (55.1)	43 (44.8)	
Comorbidities				
Diabetes mellitus	51 (29.3)	27 (34.6)	24 (25.0)	.182
Hypertension	80 (46.0)	34 (43.6)	46 (47.9)	.467
Cardiovascular disease	30 (17.2)	12 (15.4)	18 (18.8)	.687
Pulmonary disease	14 (8.0)	7 (9.0)	7 (7.3)	.782
Cerebrovascular disease	10 (5.7)	3 (3.8)	7 (7.3)	.515
Chronic kidney disease	14 (8.0)	5 (6.4)	9 (9.4)	.581
Maintenance hemodialysis	11 (6.3)	4 (5.1)	7 (7.3)	.756
Signs and symptoms				
Fever	114 (65.5)	53 (67.9)	61 (63.5)	.631
Cough	113 (64.9)	49 (62.8)	64 (66.7)	.634
Fatigue	74 (42.5)	34 (43.6)	40 (41.7)	.878
Shortness of breath	110 (63.2)	54 (69.2)	56 (58.3)	.871
Myalgia	30 (17.2)	12 (15.4)	18 (18.8)	.687
Diarrhea	21 (12.1)	9 (11.5)	12 (12.5)	.999
Medication				
Abidol tablets (200 mg *t.i.d*.)	174 (100)	78 (100)	96 (100)	1.000
Methylprednisolone sodium succinate (1–2 mg/kg/d injection for 3–5 days)	58 (33.3)	28 (35.9)	30 (31.3)	.523
Lianhua Qingwen (4 capsules t.i.d.)	171 (98.3)	76 (97.4)	95 (99.0)	.588
Mortality	29 (16.7)	19 (23.1)	10 (11.5)	.023
%SaO_2_ on admission, median (IQR)	95.0 (90.0–97.0)	95.0 (88.5–97.2)	95.0 (90.0–97.0)	.498

Abbreviations: IQR, interquartile range; SaO_2_, arterial oxygen saturation; TG, triglycerides; t.i.d., three times a day.

**Table 2:** Comparison of laboratory parameters of COVID‐19 patients

		Median (IQR)			
Variables	Normal range	Total (n = 174)	High TG (n = 78)	Control (n = 96)	*p*‐value
TG (mmol/L)	<1.7	1.6 (1.1–2.1)	2.2 (1.8–2.7)	1.1 (1.0–1.3)	<.001
Fasting blood glucose (mmol/L)	4.11–6.05	5.7 (5.1–7.1)	6.0 (5.3–7.9)	5.6 (5.0–6.5)	.321
HbA1c (%)	4.0–6.0	6.4 (5.8–7.2)	6.5 (6.0–7.4)	6.2 (5.8–6.6)	.069
Leukocytes (×10^9^/L)	3.5–9.5	5.0 (4.1–6.1)	5.1 (4.3–6.5)	4.9 (3.8–5.9)	.115
Lymphocytes (×10^9^/L)	1.1–3.2	0.8 (0.5–1.4)	0.9 (0.6–1.5)	0.8 (0.5–1.3)	.066
Serum ferritin (µg/L)	30.0–400.0	654.7 (348.5–1400.7)	798.5 (391.4–2093.8)	566.0 (291.8–1153.9)	.001
CK (U/L)	<190	73.0 (47.0–128.0)	77.5 (45.5–126.3)	69.0 (48.0–136.0)	.774
LDH (U/L)	135.0–225.0	286.0 (234.8–425.8)	302.0 (238.0–473.5)	278.0 (234.0–373.0)	.042
CRP (mg/L)	<1.0	31.0 (5.3–100.1)	39.8 (4.6–140.8)	27.9 (7.3–67.5)	.035
IL‐1β (pg/mL)	<5.0	5.0 (5.0–5.0)	5.0 (5.0–5.0)	5.0 (5.0–5.0)	.727
IL‐2 (U/mL)	223.0–710.0	714.0 (440.0–714.0)	775.5 (421.5–1267.5)	658.0 (440.0–1140.0)	.281
IL‐6 (pg/mL)	<7.0	15.3 (4.0–54.8)	20.4 (4.5–101.9)	13.0 (3.4–40.5)	.583
IL‐8 (pg/mL)	<62.0	12.9 (6.7–29.4)	10.8 (6.4–22.5)	12.8 (7.3–28.6)	.056
IL‐10 (pg/mL)	<9.1	5.0 (5.0–7.6)	5.0 (5.0–10.3)	5.0 (5.0–6.2)	.045
TNF‐α (pg/mL)	<8.1	9.2 (6.1–13.7)	9.2 (5.8–14.0)	9.2 (6.4–13.7)	.577
ESR (mm/h)	0.0–15.0	34.5 (17.0–62.0)	33.0 (20.0–58.0)	35.0 (17.0–63.0)	.659
Fbg (g/L)	2.0–4.0	5.2 (4.2–6.5)	5.2 (3.9–6.6)	5.3 (4.3–6.5)	.899

Abbreviations: CK, creatine kinase; CRP, C‐reactive protein; ESR, erythrocyte sedimentation rate; Fbg, fibrinogen; HbA1c, glycosylated hemoglobin; IL, interleukin; IQR, interquartile range; LDH, lactate dehydrogenase; TG, triglycerides; TNF‐α, tumor necrosis factor‐α.

**Figure 1:**
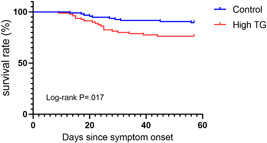
Comparison of survival rate between high TG group and control group. TG, triglycerides

SaO_2_ without oxygen inhalation on admission was 95% (IQR: 90–97%) for all patients; 95.0% (88.5–97.2%) and 95.0% (90.0–97.0) in the high TG and control groups, respectively. There was no significant difference between the groups. Overall, 29 patients (16.7%) died during hospitalization, including 19 (23.1%) in the high TG group and 10 (11.5%) in the control group (absolute survival difference, 2.5% [95% CI: 1.2‐5.1%], log‐rank *χ* ^2^ = 5.7, and *p* = .017; Table 1 and Figure 1). All deceased patients were previously treated with mechanical ventilation.

Laboratory tests showed that the TG level was 1.6 (IQR: 1.1‒2.1) mmol/L for all patients; 2.2 (IQR: 1.8‒2.7) mmol/L and 1.1 (IQR 1.0–1.3) mmol/L in the high TG and control groups, respectively. Serum ferritin, LDH, CRP, and IL‐10 levels were significantly higher in the high TG group than in the control group (*p* < .05; Table 2).

**Figure 2:**
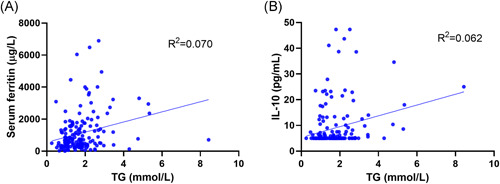
Correlation between TG level and serum ferritin, and interleukin‐10 levels. TG, triglycerides

Pearson correlation analysis showed that the TG level was positively correlated with fasting blood glucose, leukocyte, serum ferritin, LDH, CRP, and IL‐10 levels (Table 3). To solve the problem of multicollinearity among related factors, we included them in multiple linear regression equations. The results showed that the factors influencing TG were serum ferritin and IL‐10 levels (*R* ^2^ = .095; Figure 2).

**Table 3:** Pearson correlation analysis of TG in COVID‐19 patients

	Pearson's *r*	*p*‐value
Fasting blood glucose	.156	.043
Leukocytes	.198	.009
Serum ferritin	.264	.001
LDH	.208	.006
CRP	.189	.014
IL‐10	.249	.001

Abbreviations: CRP, C‐reactive protein; IL‐10, interleukin‐10; LDH, lactate dehydrogenase.

## 4 DISCUSSION

This study showed that patients with elevated TG levels had a higher mortality rate than those with normal levels. TG is degraded by lipoprotein lipase to produce free fatty acids, which can activate the nuclear factor‐κB pathway, leading to the high expression level of various proinflammatory cytokines, including TNF‐α, IL‐1β, IL‐6, and monocyte chemoattractant protein‐1.^9^ Elevated levels of triglyceride‐rich particles in the bloodstream may induce local inflammation and the activation of complement and coagulation cascades, ultimately promoting endothelial dysfunction.^10^, ^11^

In addition, TG may be related to the excessive activation of macrophages that causes the deterioration of COVID‐19 patients. In this study, regression analysis showed that TG levels were positively correlated with serum ferritin and IL‐10 levels. In the course of inflammatory diseases, infections, and malignant diseases, macrophages expressing CD163 obtain iron by clearing hemoglobin. As a result, the synthesis of ferritin in the cells increases, and its release into the blood also increases.^12^, ^13^ IL‐10 is derived from a variety of cells and is considered to be an inhibitor of inflammation and immunity. It can regulate the metabolism of macrophages by inhibiting the mammalian target of rapamycin complex 1.^14^ Many studies, including pathological studies, have shown that macrophage overactivation plays a key role in the inflammatory response and resulting organ damage in patients with severe COVID‐19.^15^, ^16^ Activated macrophages can inhibit lipoprotein lipase production to increase TG levels by releasing TNF‐α and IL‐1.^13^ Therefore, we have reason to believe that the positive correlation between TG level, serum ferritin, and IL‐10 levels in patients with COVID‐19 may be due to macrophage activation, with the level of TG representing the degree of macrophage activation.

TG levels are affected by insulin resistance‐related diseases (such as type 2 diabetes and impaired fasting glucose), drugs, and diet.^7^ Unfortunately, we did not evaluate insulin resistance by plasma insulin, HOMA‐R, and other indicators. We excluded familial hyperlipidemia, thereby also excluding the effects of hydroxychloroquine, lopinavir, ritonavir, and lipid‐lowering drugs on TG, and 75.9% of the participants were tested for HbA1c. Pearson correlation analysis showed that the TG level was correlated with fasting blood glucose level (*r* = .156, *p* = .043) but multivariate regression analysis did not show this correlation.

### 4.1 Study strength and limitations

This study provides hints that TG metabolism is involved in the pathogenesis of COVID‐19 from a clinical perspective, suggesting that this approach can be targeted for prognosis and treatment. All the patients in this study were hospitalized before the global epidemic of COVID‐19. At that time, there was no effective intervention except oxygen therapy. The pathophysiological characteristics were more easily revealed in these patients as the natural progression of the disease could be analyzed in the absence of additional therapies. In other words, the lack of effective medication at the time presented with a unique opportunity in this small sample size retrospective research to reveal the relationship between TG and prognosis, serum ferritin, and IL‐10.

This study has limitations. First of all, this is a small sample size retrospective study conducted in a single geographical area Therefore, the results should be considered as preliminary findings. In particular, the small number of deaths leaves uncertainty whether elevated TG is an independent risk factor for death in COVID‐19 patients. Second, due to special circumstance, these patients could not be followed up long‐term outside the hospital. The follow‐up data is necessary to explore the relationship between inflammation and TG. Thus, larger trials with more prognostic indicators and long‐term follow‐up are needed to determine the relationship between TG and prognosis.

## 5 CONCLUSION

COVID‐19 patients with above normal TG levels have a higher mortality rate. The TG level in COVID‐19 patients is correlated to serum ferritin and IL‐10 levels, which reflects the activation of macrophages. It is suggested that COVID‐19 patients be monitored for elevated TG as both a prognostic indicator and potential therapeutic target for COVID‐19.

## AUTHOR CONTRIBUTIONS

Peng Zhong and Zhenzhou Wang designed the study, wrote and revised the manuscript. Zhe Du reviewed the manuscript. All authors read and approved the final manuscript.

## CONFLICT OF INTERESTS

The authors declare that there are no conflict of interests.

## ETHICS STATEMENT

Ethical clearance for this study was granted by the Ethic Committee of Peking University People's Hospital. Because only the medical records were reviewed, this case series was exempted from signing the informed consent.

## Data Availability

The data sets are available from the corresponding author on reasonable request.
